# DNA Nanostructure Deposition on Self-Assembled Monolayers

**DOI:** 10.1021/acs.langmuir.5c00048

**Published:** 2025-04-28

**Authors:** Anumita Kumari, Jason Smith, Jonathan Cho, Haitao Liu

**Affiliations:** † Department of Chemistry, University of Pittsburgh, Pittsburgh, Pennsylvania 15260, United States; ‡ Department of Chemistry, Duquesne University, Pittsburgh, Pennsylvania 15282, United States

## Abstract

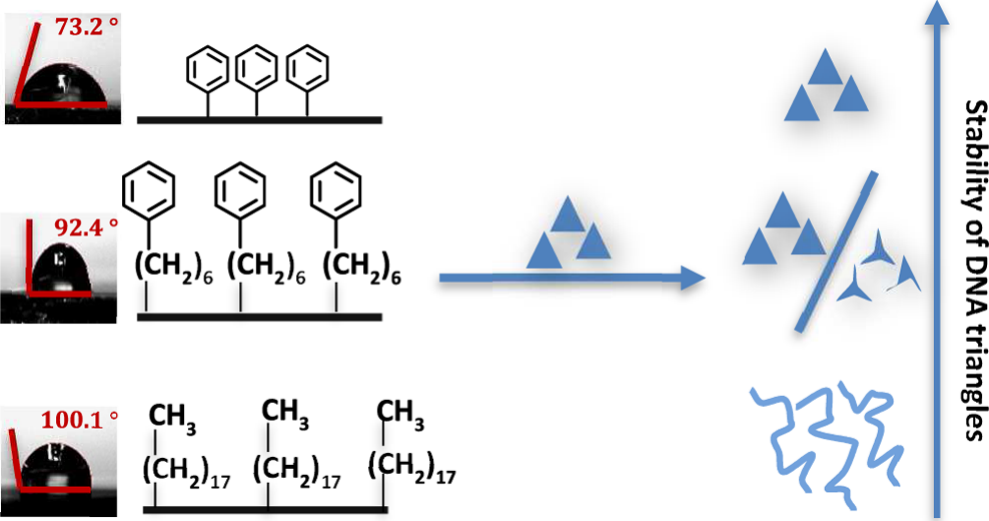

We report the deposition of DNA nanostructures on self-assembled monolayers (SAMs), focusing on the stability of DNA nanostructures on both hydrophilic and hydrophobic SAMs. Our study reveals distinct outcomes based on the nature of the SAMs. DNA nanostructures maintain structural integrity on hydrophilic SAMs, whereas they experience deformation on the most hydrophobic SAMs. Interestingly, the stability of DNA nanostructures is also sensitive to postdeposition washing procedures. The observations shed light on the intricate interplay between the wettability of SAMs and the structural stability of the DNA nanostructures. An empirical trend emerged where increased hydrophobicity is associated with a more severe deformation of DNA nanostructures. This deformation is hypothesized to arise from disrupted hydrogen bonding within DNA nanostructures and is exacerbated by interfacial tension during the drying process. Our study also highlights the potential role of π–π stacking interactions between the DNA bases and the SAMs in stabilizing the DNA nanostructures. Our work expands the type of substrates that can be used for applications of DNA nanotechnology and highlights the need for a comprehensive understanding of the interactions between DNA nanostructures with different surfaces.

## Introduction

DNA nanostructures play a unique role in supramolecular chemistry, offering unparalleled capabilities for precise functionalization and patterning at the nanoscale.^1^ These structures can be made into designer shapes and further tailored with functional groups at predetermined locations. As a result, DNA nanostructures can serve as a foundation for creating intricate surface patterns or arrays with nanoscale accuracy. Its programmable nature allows for molecular recognition, making it invaluable in sensor applications where specific molecule detection is crucial.^2^ Furthermore, DNA nanostructures can act as a template, guiding the assembly of other molecules or nanostructures on surfaces.^3^ Collectively, these attributes position DNA nanostructures as a transformative tool in advancing surface chemistry research and applications.

Many applications of DNA nanostructures require their deposition on a solid substrate.^3−6^ This substrate has always been hydrophilic in nature, as very little is understood about the interaction of DNA nanostructures with hydrophobic surfaces. Given the wide range of hydrophobic surfaces used in research and development, understanding their interaction with DNA nanostructures may lead to many new applications. Studies investigating the stability of charged molecules on hydrophobic surfaces have shown that their ultimate shape is determined by a competition between adsorption energy gain and elastic free energy penalty.^7^ Lower cross-linking in molecules leads to greater deformation as they attempt to minimize unfavorable interactions between water and the hydrophobic surface.^8^ Similarly, hydrophobic substrates, which prefer to avoid contact with water, promote significant deformation of adsorbed molecules to maximize contact with the surface.^7^ In the case of dsDNA, interactions with hydrophobic surfaces such as highly oriented pyrolytic graphite (HOPG) result in molecular overlap and structural rearrangement due to “sticky-ended” cohesions, leading to conformation changes and DNA–DNA interactions.^9^ Molecular simulations further reveal that hydrophobic surfaces strengthen terminal DNA base pair hydrogen bonds while destabilizing central base stacking, influencing DNA hybridization and structural integrity.^10^ These findings suggest that DNA nanostructures on hydrophobic SAMs may undergo significant deformation due to similar energetic considerations, further highlighting the complexity of DNA-hydrophobic surface interactions.

We are interested in studying the deposition of DNA nanostructures on SAMs due to their versatility in modifying surfaces.^11^ Discovered by Jacob Sagiv in 1980, SAMs have been extensively researched and can be formed on many substrates, including SiO_2_/Si.^12^ SAM has found applications in diverse areas such as biosensors^13,14^ and microelectronics.^15,16^ In biosensing, the use of spacer-thiolated molecules and thiolated ssDNA to form binary SAMs has proven to be effective in enhancing detection sensitivity. This approach was successfully employed to detect polymerase chain reaction (PCR) amplicons from the genomic DNA of *Escherichia coli* K12, with a detection limit as low as 60 fg of DNA (equivalent to 10 copies).^17^ In microelectronics, DNA-based self-assembly has enabled the precise positioning of single biological molecules on solid-state devices with nanoscale resolution. We believe that combining DNA nanostructures with SAMs could allow multipoint anchoring of biomolecules with tunable binding strength, configuration, and density on a broad range of substrates.^18^ Despite the promising interactions between biomolecules and various SAMs, most studies have focused on hydrophilic ones.^19−22^ The interaction between DNA nanostructures and hydrophobic SAMs remains a relatively uncharted territory.

Herein, we report the structural stability of DNA nanostructures deposited on hydrophilic and hydrophobic silane SAMs prepared on SiO_2_/Si. We found that the stability of DNA nanostructures decreases with an increase in the hydrophobicity of SAMs and that π–π interactions with the surface can facilitate the adsorption of DNA nanostructures on relatively hydrophobic substrates. We propose that the washing procedure after deposition is crucial for maintaining the structural integrity of DNA nanostructures on hydrophobic substrates.

## Experimental Section

### Materials and Methods

Silicon wafers (625 ± 15 μm) with native oxide layers were purchased from Vishay Siliconix Inc. M13mp18 scaffold (Bayou Biolabs) and synthetic staple DNA stands (Integrated DNA Technologies) were used to fabricate the DNA nanostructures. Buffer solution for DNA was made from Tris-acetate-EDTA (TAE) buffer and magnesium acetate. The piranha solution was made from sulfuric acid (H_2_SO_4_) and hydrogen peroxide solution (30% H_2_O_2_) purchased from Sigma-Aldrich. SAMs were created from octadecyltrichlorosilane (OTCS, ≥99.5%), 3-aminopropyl-trimethoxysilane (APTES), 6-phenylhexyltrichlorosilane (PHTCS), and phenyltrichlorosilane (PTCS, ≥95%). Methanol (≥99.8%), ethanol (≥99.5%), hexane (mixture of isomers, ≥98.5%), toluene (≥99.5%), chloroform (≥99.5%), and 2-propanol (≥99.5%) were purchased from Millipore Sigma. Sodium chloride (≥99.0%) and all other chemicals were purchased from Thermo Fisher Scientific.

### DNA Nanostructure Synthesis

The design and synthesis of the DNA triangle structure was previously reported.^23^ Briefly, a solution of 180 μL of TAE/Mg^2+^ buffer [Tris (40 mM, pH 8.0), acetic acid (20 mM), EDTA (1 mM), and Mg acetate (12.5 mM)], 8.6 μL of M13mp18 ssDNA (1 μg/mL), 15 μL of 256 short staple strands (300 nM), and 77 μL of ultrapure water were mixed and separated into 4 Eppendorf tubes equally. The tubes were placed in an MJ Research Minicycler and cooled from 95 to 20 °C at the rate of 1 °C/min. After the cooling, the solution from the four tubes were combined into a single 30K Omega Nanosep centrifugal filter, centrifuged in a benchtop single speed microcentrifuge (VWR Galaxy Ministar) to remove the excess staple strand, and additional TAE/Mg^2+^ buffer was added to the remaining liquid. The centrifugation and addition of new buffer was repeated another 2 times. The final concentration of DNA triangle was determined by using UV-Vis spectroscopy. The concentration of the DNA solution was between 20 and 40 μg/mL. The final solution was placed in a refrigerator.

### Cleaning the Silicon Wafer

The silicon wafer was first sonicated in deionized water for 30 min. Then the wafer was cleaned in piranha solution (70:30 H_2_SO_4_/H_2_O_2_) for 30 min at 65 °C. (Warning: hot piranha solution is extremely dangerous and volatile; it reacts violently with organic materials; do not leave unattended or exposed to extreme heat.) The wafer was thoroughly washed with ultrapure water and dried with N_2_ gas. Immediately following this washing, the wafer was baked in an oven for 30 min at 100 °C to remove any remaining water.

### Assembly of SAM on the Cleaned Silicon Wafer

For the growth of OTCS, PTCS, PHTCS, or APTES SAM, the cleaned Si wafer was removed from the oven and immediately placed in an air-free glovebox. The wafer was exposed to a 1 mM SAM solution prepared with anhydrous toluene for various amounts of time. We used measurements of film thickness (Table S1) and water contact angle (Table S2) to confirm the formation of the SAMs. It was found that to prepare SAM for PTCS, PHTCS, and APTES, keeping the wafer in 1 mM silane solution for an hour was enough, consistent with the literature.^24−26^ Following similar study on OTCS SAM formation, the wafer was exposed to 1 mM OTCS solution for 9 h.^27,28^ The wafer was then rinsed thoroughly with excess anhydrous toluene and baked in an oven at 120 °C for 5 min. The wafer was then washed with isopropanol and sonicated in chloroform for 30 min. Finally, the wafer was washed with 2-propanol and dried with N_2_ gas.

### Film Thickness and Wettability of SAM

For measuring the film thickness, an Alpha-SE Ellipsometer with an incidence angle of 65° was used. Two program models were used to measure the film thickness of the samples. The first model, “NTVE_JAW”^29^ was used for the native oxide layer, while a Cauchy model^30^ was used for the SAM film. Film thickness was measured at five different spots on the wafer, and then the average of those five measurements was taken. For wettability measurements, a VCA Optima contact angle tester was used. Three measurements were taken for each wafer, and then the average of those three was reported.

### Deposition of the DNA Nanostructure on Silicon and SAM Wafers

The wafer was placed in a Petri dish, and 20 μL of a 5 μg/mL solution of DNA nanostructure was deposited on its surface. The Petri dish was then covered with a wet paper towel to maintain a humid environment. The wafer was allowed to sit for 30 min and then thoroughly washed in 90:10 ethanol: water solution and dried with N_2_ gas (in case of Si and APTES). For PTCS, PHTCS, and OTCS SAM, after the deposition of DNA suspension, the wafer was allowed to sit for 30 min and then blown dry with N_2_.

### Topographical Analysis of Wafers with Atomic Force Microscopy

Wafers were analyzed using an Asylum MFP3D atomic force microscope. Cross sections were taken to identify the height of the nanostructures on the silicon and SAM wafers. These cross sections were then analyzed by using Asylum AR 16.19.220 software. For wafers with DNA deposition, both the center and edge of the wafer was analyzed. This was to determine whether the distribution and condition of the DNA nanostructures are uniform across the entire surface.

### Quantitative Analysis of DNA Nanostructures Stability on SAMs

Three APTES-coated wafers were incubated with 20 μL of a 5 μg/mL DNA nanostructure solution for 30 min, washed 4–5 times in 90/10 ethanol/water, N_2_-dried, and imaged by AFM. Two images per wafer were analyzed to quantify the structural integrity of DNA nanostructures.

For quantitative analysis of DNA nanostructure stability on PTCS SAM, nine wafers were prepared with PTCS and each covered with 20 μL of a 5 μg/mL DNA nanostructure solution. Three wafers were N_2_-dried and imaged via AFM. Three were dipped in an alcohol/water mixture, N_2_-dried, and imaged via AFM. The final three underwent five dipping cycles before N_2_-drying and imaging. Two AFM images per wafer were analyzed for intact vs deformed DNA triangles. For quantitative analysis of DNA nanostructure stability on PHTCS SAM, ten PHTCS-coated wafers were incubated with 20 μL of a 5 μg/mL DNA nanostructure solution for 30 min, washed in 90/10 ethanol/water, N_2_-dried, and imaged via AFM. One image per wafer was analyzed for intact vs deformed DNA triangles.

### Stability of DNA Nanostructures in Organic Solvents

SAM wafers with DNA nanostructures were immersed in hexane, ethanol, or toluene solvents for 1 and 2 h. The wafers were then dried with N_2_ gas.

### Stability of DNA Nanostructures in Deionized Water

SAM wafers with DNA nanostructures were immersed in deionized water for 10 s, 5 min, or 1 h. The wafers were then dried with N_2_ gas.

### Influence of Ionic Strength on the Stability of DNA Nanostructures

SAM wafers with DNA nanostructures were immersed in a NaCl solution of desired concentration (0.01–0.2 M) for 10 s. The wafers were then dried with N_2_ gas. Depending on surface cleanliness, most of the samples were washed once or twice in the 9/1 (v/v) ethanol/water solution for 3 s to remove any potential salt residues. The washed wafers were then dried using N_2_ gas before AFM imaging.

## Results and Discussion

### SAM Characterization

**Figure 1:**
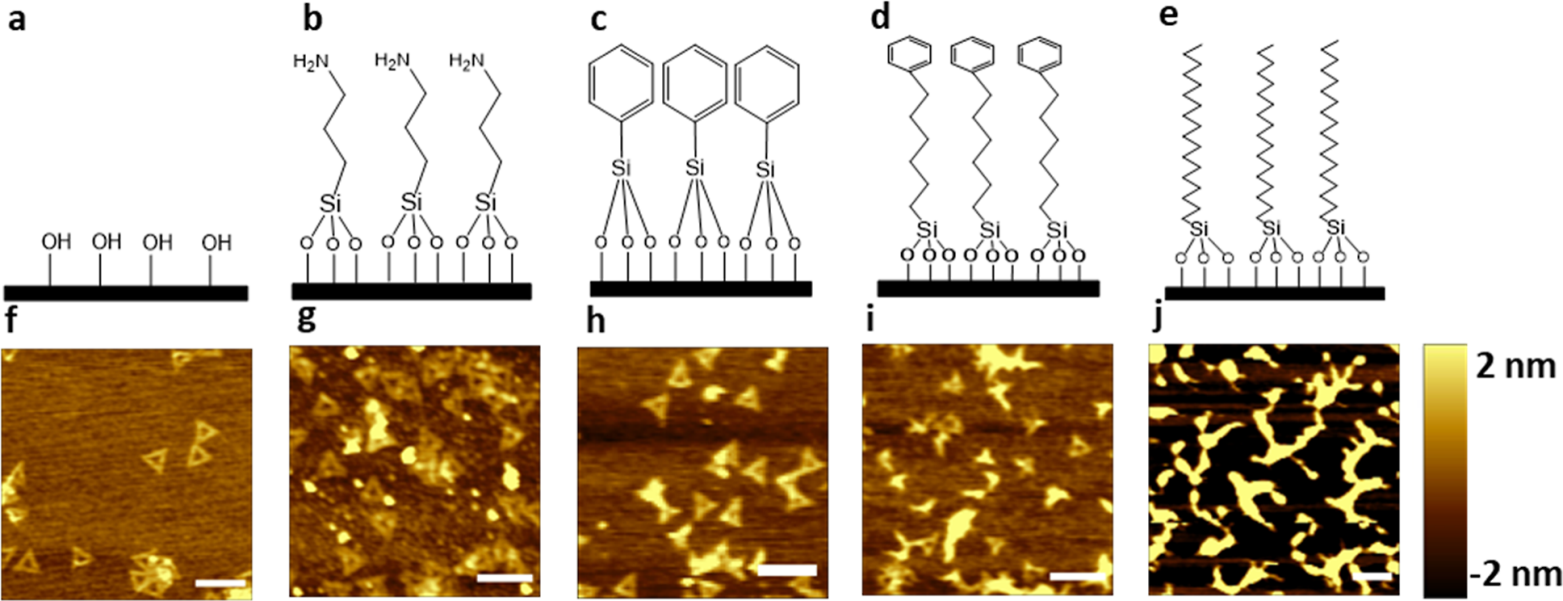
Deposition of the DNA nanostructure on the Si wafer with various terminal functional groups. (a) Hydroxyl functional group on the Si wafer, (b) amine functional group on the APTES SAM, (c) phenyl functional group on PTCS SAM, (d) phenyl functional group on the PHTCS SAM, and (e) methyl functional group on the OTCS SAM. AFM image of DNA nanostructures deposited on Si wafers (f) without SAM and with SAMs, (g) APTES, (h) PTCS, (i) PHTCS, and (j) OTCS. The scale bars represent 200 nm. The Si wafer is shown as a black plate in (a–e).

As seen in Figure 1, a variety of SAM end groups were chosen for this study. We characterized the SAM by measuring the water contact angle and film thickness (Supporting Information, Tables S1 and S2). The SAMs chosen for this study have water contact angles ranging from ca. 54° (hydrophilic) to 100° (hydrophobic). The SAM with the most hydrophilicity is (3-aminopropyl) triethyloxysilane (APTES, Figure 1b) with a water contact angle of 54.1 ± 0.5°, followed by phenyltrichlorosilane (PTCS, Figure 1c) with a water contact angle of 73 ± 1.4° and 6-phenylhexyltrichlorosilane (PHTCS, Figure 1d) with a water contact angle of 92.4 ± 0.5°. SAM with the highest hydrophobicity was octadecyltrichlorosilane (OTCS, Figure 1e) with a water contact angle of 100.1 ± 0.9°.

### DNA Nanostructure Deposition on SAMs

**Figure 2:**
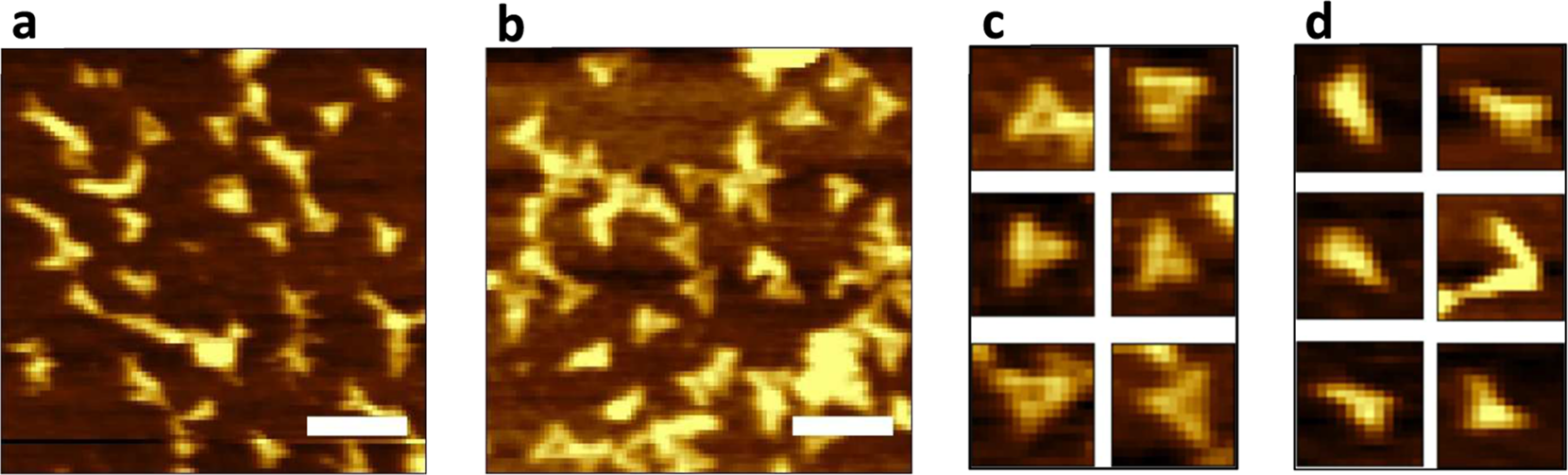
AFM images of DNA nanostructures deposited on PHTCS SAM showing dual outcomes: (a,d) deformed and (b,c) intact DNA nanostructures. The scale bars represent 200 nm.

**Figure 3:**
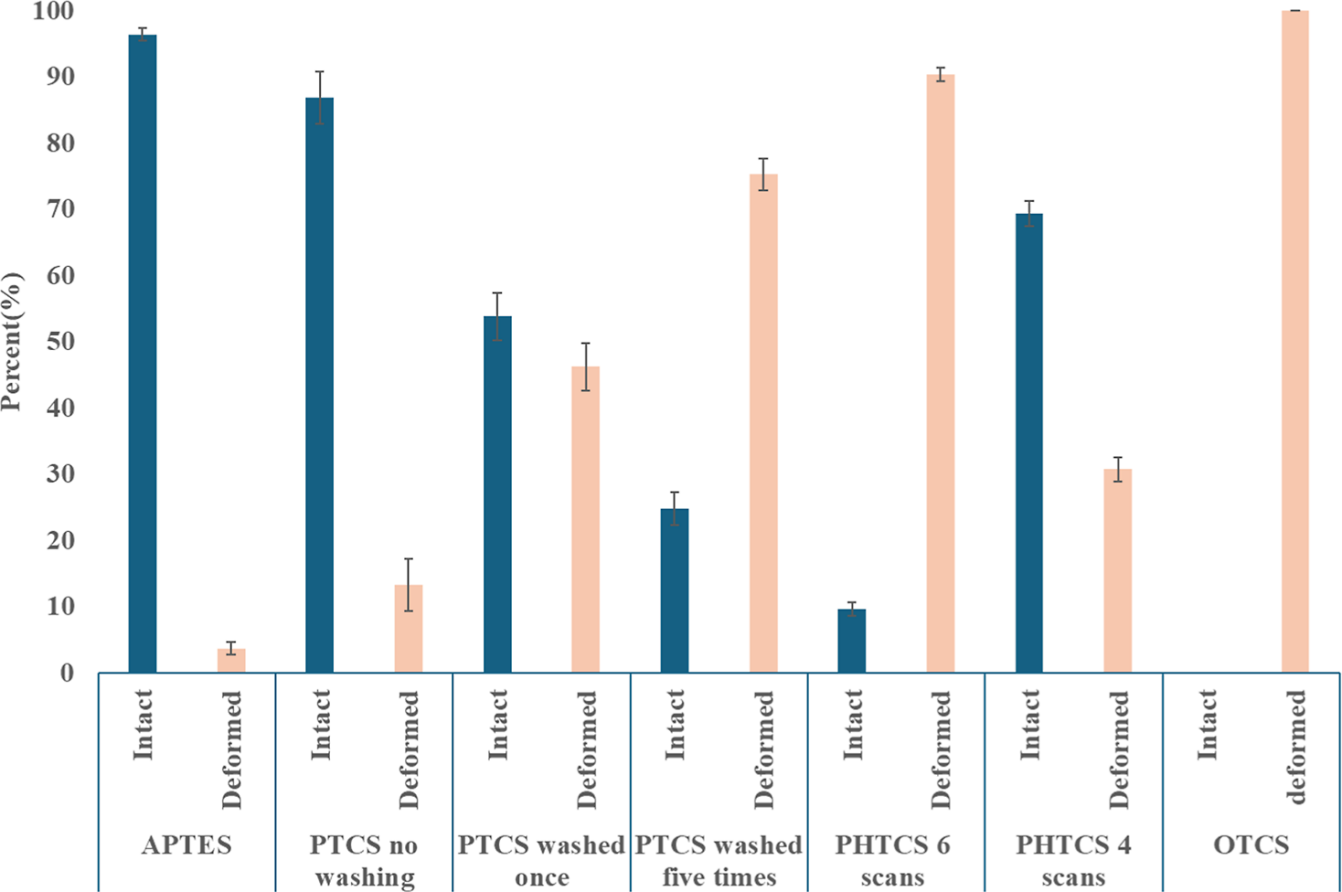
Percentage of intact and deformed DNA nanostructures on SAMs.

We observed very different outcomes after the deposition of DNA nanostructures on the SAM substrates. We deposited an equal volume of DNA nanostructures (5 μg/mL) on each substrate, and after 30 min, we washed the wafer with 90/10 (v/v) ethanol/water mixture. The morphology of the DNA nanostructure was examined using an atomic force microscope (Figure 1). Intact DNA triangles were observed on Si/SiO_2_, APTES, and PTCS wafers (Figure 1f–h). For PHTCS, we observed both intact and deformed DNA nanostructures (Figures 1i and 2); while for OTCS, we consistently observed deformed DNA nanostructures (Figure 1i). Percentage of intact and deformed DNA structures for all samples are summarized in Figure 3.

Si wafers are commonly used substrates and are used as a reference in our study. The interaction between Si wafer and DNA is well understood.^31,32^ The deposition of the DNA nanostructure on the Si wafer is assisted by the formation of a Mg^2+^ salt bridge mediating the electrostatic interaction between the negatively charged wafer surface and the DNA backbone. For APTES, being a hydrophilic and positively charged SAM under our deposition conditions (pH = 8), we expected strong electrostatic interaction with DNA nanostructures where the positive charges on the SAM would attract and stabilize the DNA nanostructures, providing the necessary adhesion to the surface and preventing it from being deformed.

We observed two very different experimental outcomes for the samples deposited onto PHTCS SAMs: intact (Figure 2b,c) and deformed (Figure 2a,d) DNA nanostructures. In six out of ten trials, we observed that more than 90% of DNA nanostructures completely lost their structural integrity, while in the remaining four trials, we observed 30% deformed and 70% intact DNA nanostructures (Figure 3). This result with PHTCS is notably different from that with PTCS, where most of the DNA structures were intact. The difference in the observed stability could be due to the orientation of the phenyl groups. In PTCS, the phenyl group is oriented perpendicular to the silicon surface, whereas in PHTCS, it is tilted away from the normal,^33^ potentially leading to stronger π–π interactions with the DNA nanostructures. There are literature reports of significant deformation of DNA nanostructures deposited on polystyrene,^34^ with its phenyl group tilted at 27°,^35^ as well as on HOPG,^9,36,37^ where the π system is parallel to the surface. It is possible that the orientation of the phenyl group in PHTCS SAM is sensitive to experimental conditions and even spatially heterogeneous. Additional experiments are needed to identify the underlying causes.

For the OTCS SAM, the most hydrophobic SAM in this study, we observed consistent deformation of DNA nanostructures. We deposited a 5 μg/mL DNA nanostructure on the OTCS-modified wafer. After 30 min, we blew dry the wafer using a stream of N_2_. DNA nanostructures deposited on OTCS SAM do not retain their morphology and appear to be completely stretched, as seen in Figure 1e. The DNA materials are also aggregated on the surface because the height of the nanostructure is >6 nm (Figure S1), significantly larger than the expected height of DNA triangles, which is approximately 2 nm when deposited on Si/SiO_2_.

### Effect of Postdeposition Washing Procedures

In the case of PTCS, the morphology of the DNA nanostructure is sensitive to postdeposition washing procedures. After the DNA nanostructure suspension is deposited on a surface, it is a well-documented protocol to wash the surface 2–3 times using water or ethanol/water mixture to remove excess Mg^2+^ and other salt residues from the buffer.^38^ Wafers that are not washed often show residues upon visual inspection, and AFM imaging shows undesirable coating on the DNA nanostructures and higher surface roughness. Because this protocol was developed for depositing DNA nanostructures on hydrophilic surfaces, we explored the effect of postdeposition washing on the structural stability of DNA nanostructures deposited on PTCS.

**Figure 4:**
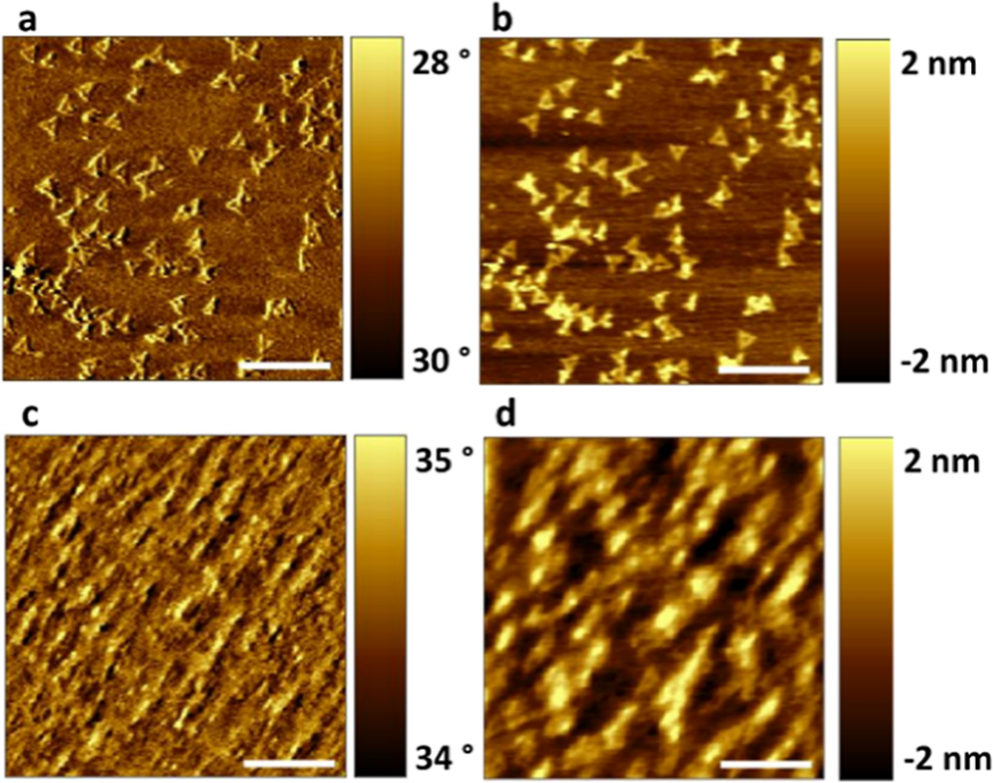
AFM height and phase images of DNA nanostructures deposited on Si wafer and PTCS SAM wafer without washing with 90:10 ethanol/water mixture. (a) Phase and (b) height images of DNA triangle deposited on PTCS SAM wafer and (c) phase and (d) height images of DNA triangle deposited on Si wafer. The scale bars represent 500 nm.

We deposited an equal volume of 5 μg/mL DNA nanostructure on the PTCS SAM and Si wafers. After 30 min, the wafers were blown dried using a stream of N_2_. Height and phase images were taken using AFM immediately after drying the wafers without additional solvent washing. It was observed that washing PTCS wafers with an alcohol/water mixture after DNA deposition is not necessary. As seen in Figure 4a,b, there was no salt residue left behind and we were able to obtain clear images of DNA triangles on PTCS SAM without postdeposition washing. In contrast, for the sample deposited on the Si wafer, we observed higher surface roughness due to salt deposit, and the phase image indicated the presence of salt on the surface (Figure 4c,d). We believe the hydrophobic nature of the PTCS substrate makes it easier to fully remove the buffer during the blow-drying process and greatly reduces the amount of salt residue.

An empirical trend observed in this study is that a higher water contact angle is associated with more severe and consistent deformation of DNA nanostructures. We hypothesize that hydrophobic SAM disrupts the hydrogen bonding within the DNA nanostructure. While DNA is considered an overall hydrophilic biomolecule, most of its hydrophilicity comes from the phosphate backbone; the bases are much more hydrophobic and can interact with hydrophobic substrates as previous studies have shown.^37^ The bases are known to interact with other aromatic molecules through π–π stacking. This interaction may result in stronger interaction with the substrate and decide the morphological stability of the DNA nanostructure, as we observed in PTCS and PHTCS.

**Figure 5:**
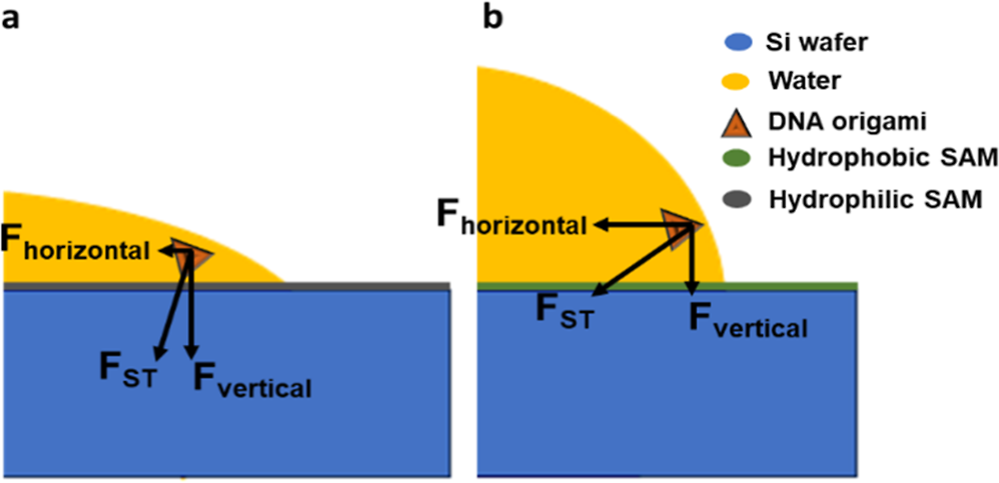
Force experienced by a DNA nanostructure deposited on (a) hydrophilic and (b) hydrophobic substrates. *F*_ST_: surface tension.

We further hypothesize that the surface tension at the solid–liquid–vapor three phase lines causes deformation and aggregation of the DNA nanostructures. As shown in Figure 5, as the solvent front moves across the SAM surface during the solvent drying process, the DNA nanostructures at the phase boundary will experience surface tension (*F*_ST_) that is perpendicular to the liquid surface and pointing into the liquid phase. The horizontal component of the surface tension increases with an increasing contact angle. Therefore, the lateral “drag” (*F*_Horizontal_ in Figure 5b) experienced by DNA nanostructures during the solvent drying process would be stronger when it is deposited on a hydrophobic surface and make it more likely to deform.

**Figure 6:**
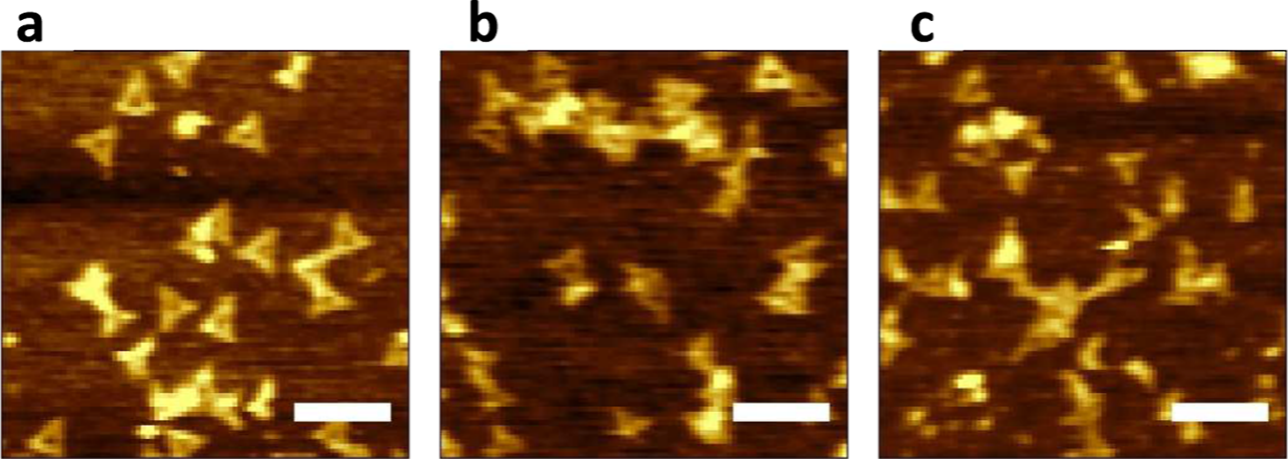
AFM image of DNA nanostructures deposited on PTCS wafer and exposed to different washing treatments: (a) no washing and (b) washed once and (c) 5 times by dipping in 90/10 (v/v) ethanol/water mixture. The scale bars represent 200 nm.

Consistent with this hypothesis, we found that postdeposition washing steps significantly impact the morphology of the DNA nanostructure on PTCS. We deposited an equal volume of 5 μg/mL DNA nanostructure on three PTCS wafers. After incubating the substrates for 30 min, one wafer was blow-dried using a stream of N_2_; the second wafer was directly dipped in a 90/10 (v/v) ethanol/water mixture for 10 s and blow-dried using N_2_; the last wafer was subjected to the dipping and drying cycle five times. AFM images were taken immediately after the final drying of all three samples. Wafers that were not washed or washed once in ethanol/water mixture had intact DNA nanostructure, as seen in Figure 6a,b. The wafers underwent 5 cycles of drying and washing and showed significant deformation of the DNA nanostructures (Figure 6c). In contrast, repeated washing and drying do not negatively impact the morphology of the DNA nanostructure when they are deposited on the Si/SiO_2_ wafer (Figure 1a). This result supports our hypothesis that DNA deposited on hydrophobic surface experiences a higher stretching force during drying, thus destabilizing its structural integrity.

### Stability of Deposited DNA Nanostructures

We investigated the stability of deposited DNA nanostructures in various solvents (Figures S2–S6). As shown in Table 1, desorption of DNA nanostructures was observed once the wafer was dipped in either water (5 min) or salt solutions (10 s) of varying concentrations (Figures S4–S6). Immersion in ethanol for 2 h resulted in the deformation of DNA nanostructures on PTCS SAM and desorption of DNA nanostructures on APTES SAM (Figures S2 and S3). For nonpolar solvents such as toluene and hexane, the DNA nanostructures deposited on APTES and PTCS SAMs remained stable even after 2 h of immersion (Figures S2 and S3).

**Table 1:** Effect of Solvent Immersion on the Stability of DNA Nanostructures Deposited on PTCS and APTES SAMs

	exposure time	DNA on PTCS	DNA on APTES
water	5 min	desorbed	desorbed
0.01 M NaCl	10 s	desorbed	desorbed
0.05 M NaCl	10 s	desorbed	desorbed
0.2 M NaCl	10 s	desorbed	desorbed
ethanol	2 h	deformed	desorbed
hexane	2 h	stable	stable
toluene	2 h	stable	stable

**Figure 7:**
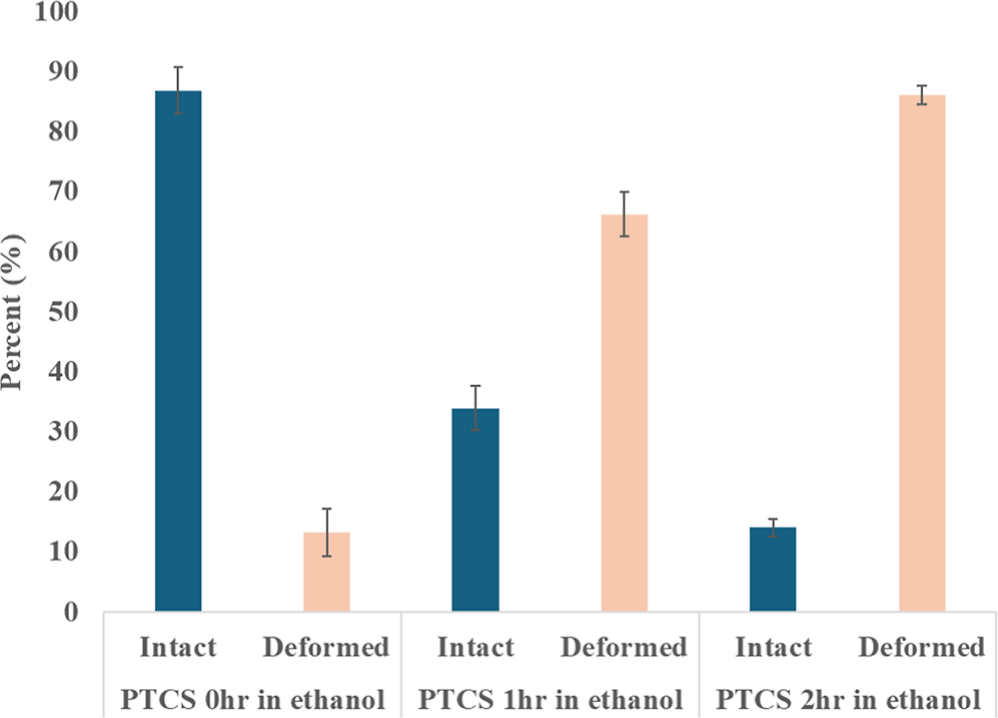
Percentage of intact and deformed DNA nanostructures deposited on PTCS SAM under various postdeposition treatments.

We also observed that the extent of deformation of DNA nanostructures deposited on PTCS SAM increases with a longer exposure to ethanol. The wafers exposed to ethanol for 1 and 2 h showed 33% and 13% of intact DNA nanostructures, respectively (Figure 7). These data are comparable to those obtained after five washing cycles using an ethanol/water mixture (24%, Figure 6c). These results suggest that polar solvents compete with nonpolar substrates for interaction with DNA-deposited nanostructures, leading to increased deformation or desorption.

## Conclusion

This study examines the structural stability of DNA nanostructures on SAMs with varying wettability. Results indicate that DNA nanostructures remain structurally intact on the relatively hydrophilic APTES and PTCS SAMs but undergo complete deformation on the hydrophobic OTCS SAM, which we attribute to disrupted hydrogen bonding and interfacial tension during solvent drying. Postdeposition washing procedures significantly impact the structural integrity of samples deposited on PTCS SAM. There is some evidence suggesting that the π–π stacking interaction may play a role in the outcome of the deposition although additional investigation is needed to fully understand its contribution. The deposited DNA nanostructures remain intact in nonpolar solvents but desorbs in water and salt solutions. Work is underway to optimize surface chemistry, drying methods, and deposition conditions to enhance the structural integrity and long-term stability of the deposited DNA nanostructures. This work provides new insights into the interactions between DNA nanostructures and surfaces and broadens the range of compatible substrates. We hope that this result will create new opportunities for DNA nanotechnology across a wider array of applications in surface engineering, biosensing, and nanoelectronics.

## Supporting Information Available

Film thickness and water contact angle of SAM, height profile of DNA nanostructures deposited on OTCS, and AFM images of DNA nanostructures on PTCS and APTES before and after exposure to polar and nonpolar solvents (PDF)
