# Supplementary material for: DNA Nanostructure Deposition on Self-Assembled Monolayers
Source: Langmuir. 2025 Apr 28;41(18):11367–73. doi: 10.1021/acs.langmuir.5c00048 (PMC12080317; doi:10.1021/acs.langmuir.5c00048)
Supplement: Supplementary file 1 — la5c00048_si_001.pdf [file la5c00048_si_001.pdf]

DNA nanostructure deposition on self-assembled monolayers

Anumita Kumari,[a ‡] *Jason Smith* [a, b‡] *, Jonathan Cho* [a] *and Haitao Liu*[a *].

[a].Department of Chemistry, University of Pittsburgh, Pittsburgh, PA, 15260.

[b].Department of Chemistry, Duquesne University, Pittsburgh, PA, 15282

*Email: hliu@pitt.edu

Supporting Information

Table S1. Film thickness (nm) of SAM on Si wafer after various growth time.

SAM	1 Hour	3 Hour	6 Hour	9 Hour	16 Hour	20 Hour	22 Hour	24 Hour
**OTCS**	1.34 ± 0.03	1.81 ± 0.08	1.93 ± 0.05	2.20 ± 0.05	3.10 ± 0.05	3.59 ± 0.03	3.61 ± 0.02	3.64 ± 0.12
**APTES**	0.82 ± 0.05	0.92 ± 0.03	1.13 ± 0.02	1.36 ± 0.01	1.43 ± 0.04	1.49 ± 0.04	1.92 ± 0.02	1.95 ± 0.03
**PTCS**	1.16 ± 0.06	1.35 ± 0.04	1.59 ± 0.05	1.89 ± 0.04	2.10 ± 0.09	2.20 ± 0.15	2.53 ± 0.12	3.12 ± 0.11
**PHTCS**	1.44 ± 0.02	1.54 ± 0.02	1.64 ± 0.02	1.92 ± 0.02	2.55 ± 0.02	3.04 ± 0.16	3.11 ± 0.11	3.18 ± 0.13

Table S2. Water contact angle of SAM grown on Si wafer for various time periods.

SAM	1 Hour	3 Hour	6 Hour	9 Hour	16 Hour	20 Hour	22 Hour	24 Hour
**OTCS**	53.2 ± 0.8°	83.7 ± 0.8°	91.9 ± 1.2°	100.1 ± 0.9°	105.3 ± 0.9°	104.3 ± 0.5°	103.4 ± 0.4°	104.1 ± 0.4°
**APTES**	42.7 ± 0.7°	54.1 ± 0.5°	57.1 ± 0.4°	60.0 ± 0.3°	63.2 ± 0.3°	63.8 ± 0.1°	63.9 ± 0.1°	64.0 ± 0.1°
**PTCS**	73.0 ± 1.4°	75.6 ± 1.5°	80.4 ± 0.7°	80.3 ± 0.9°	81.3 ± 1.2°	80.9 ± 1.4°	81.2 ± 1.2°	81.5 ± 3.2°
**PHTCS**	92.4 ± 0.1°	97.6 ± 0.2°	98.1 ± 0.6°	98.2 ± 0.5°	98.2 ± 0.2°	98.5 ± 0.5°	98.5 ± 0.6°	99.5 ± 0.4°

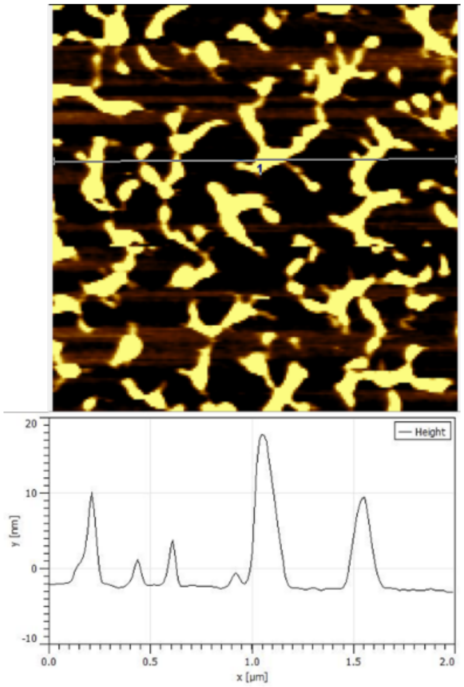

Fig S1. AFM image (top) of DNA origami deposited on OTCS SAM. The height of the structure as seen on the cross-section profile (bottom) indicates aggregation.

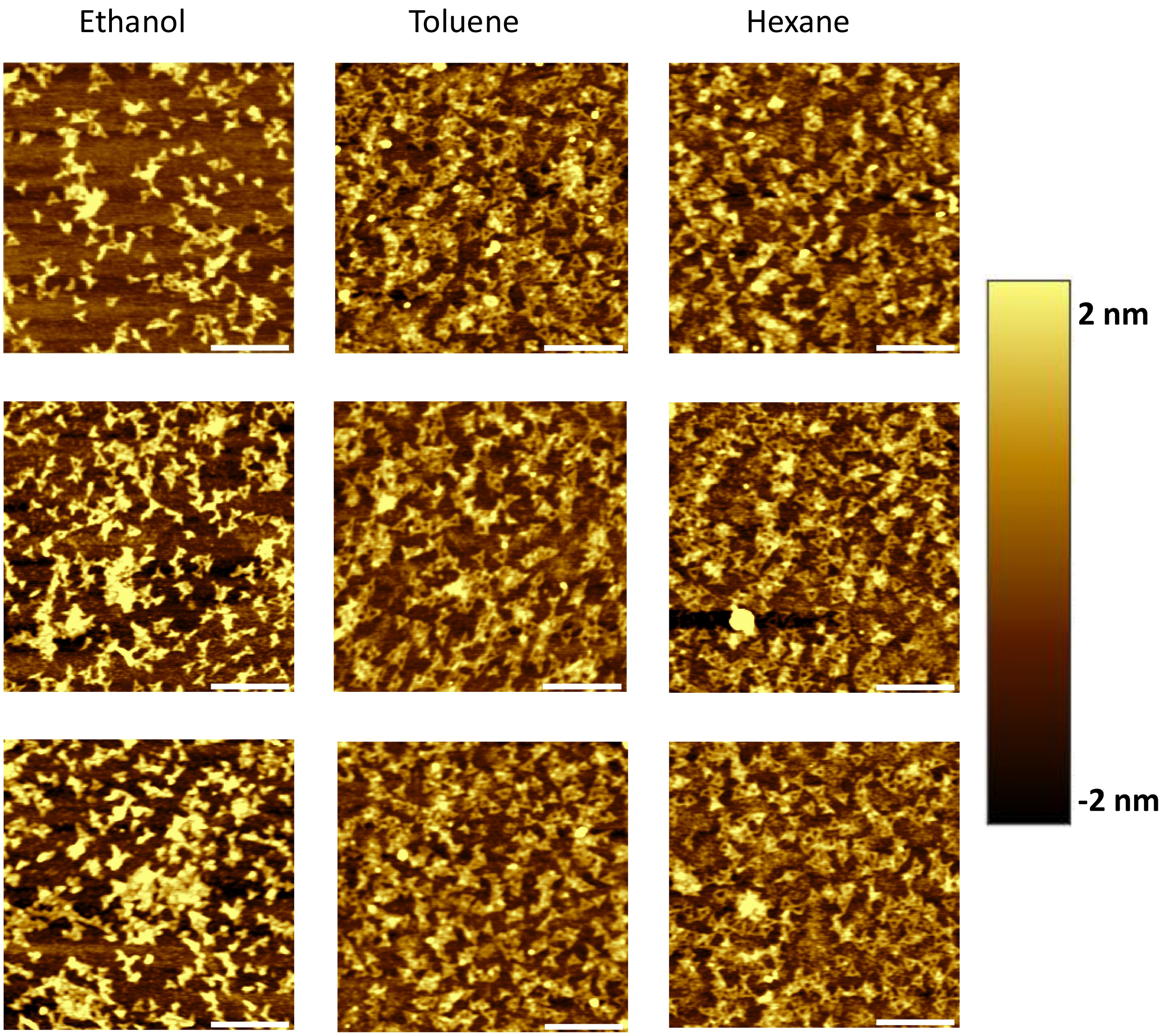

Fig S2. AFM images of DNA origami deposited on PTCS before (first row) and after exposure to ethanol (left column), toluene (middle column), and hexane (right column) for 1 hr (second row), and 2 hrs (third row). All scale bars represent 500 nm.

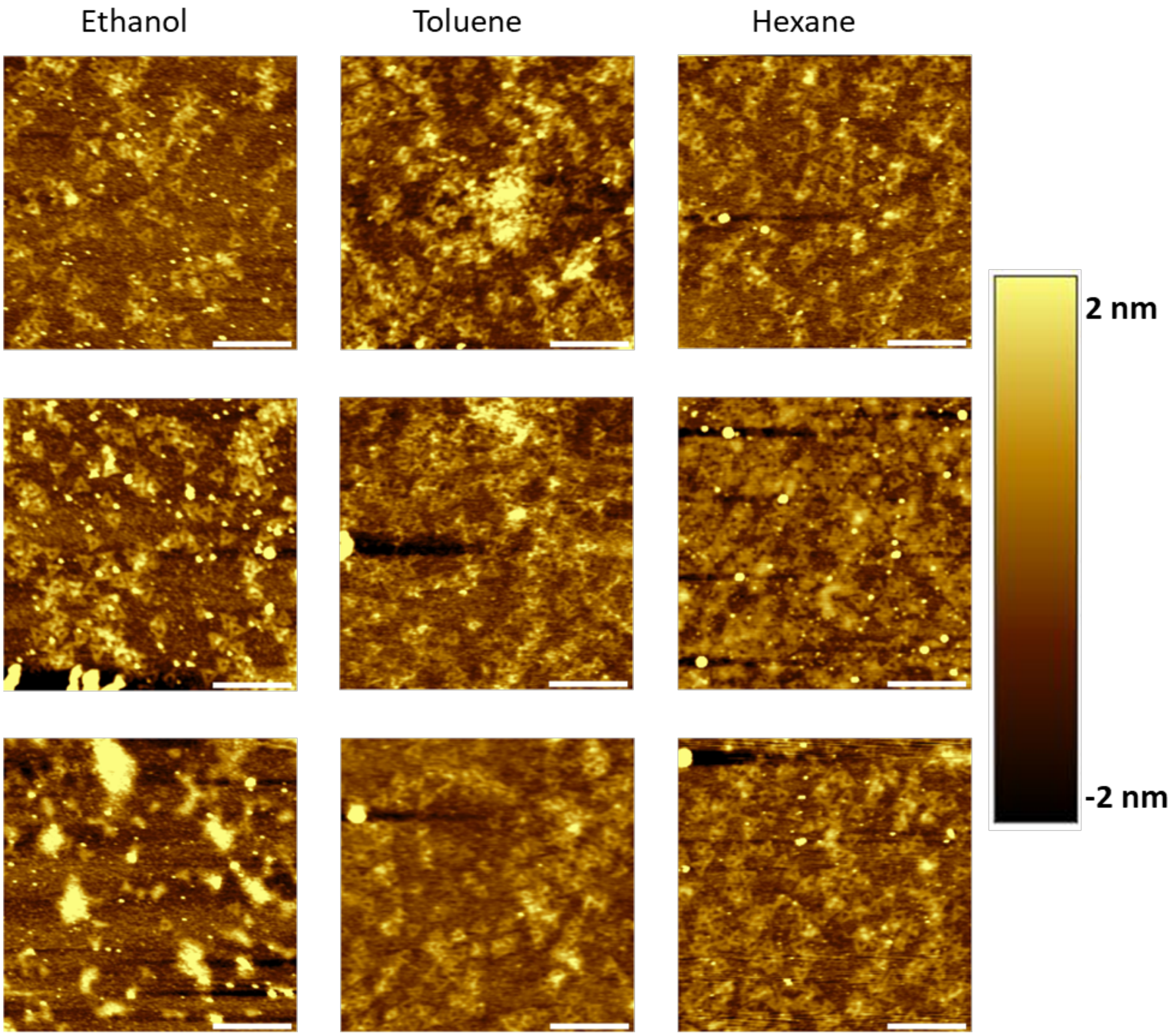

Fig S3. AFM images of DNA origami deposited on APTES before (first row) and after exposure to ethanol (left column), toluene (middle column), and hexane (right column) for 1 hr (second row), and 2 hrs (third row). All scale bars represent 500 nm.

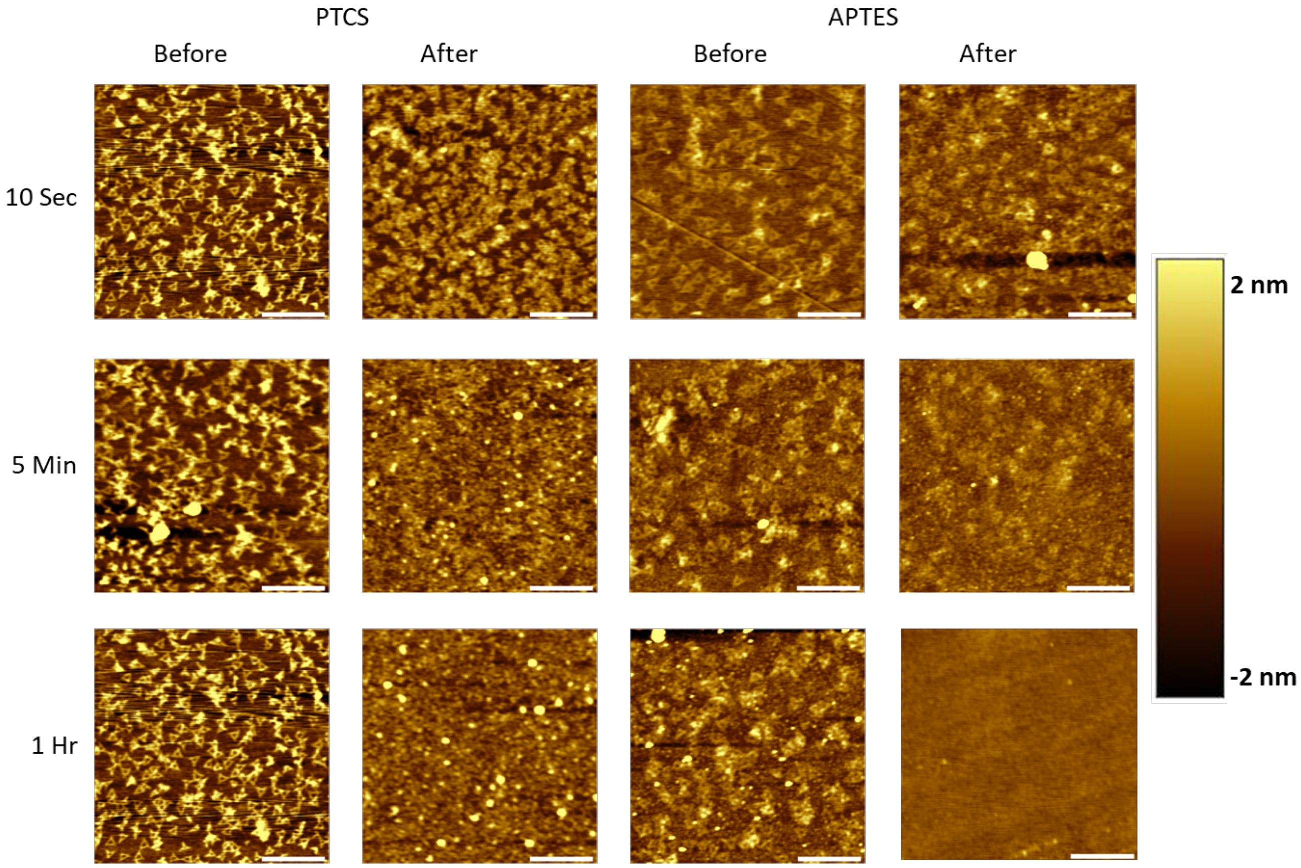

Fig S4. AFM images of DNA origami deposited on PTCS and APTES before (first column and third column respectively) and after (second column and fourth column respectively) exposure to DIW, for 10 sec (first row), 5 min (second row) and 1 hr (third row). All scale bars represent 500 nm.

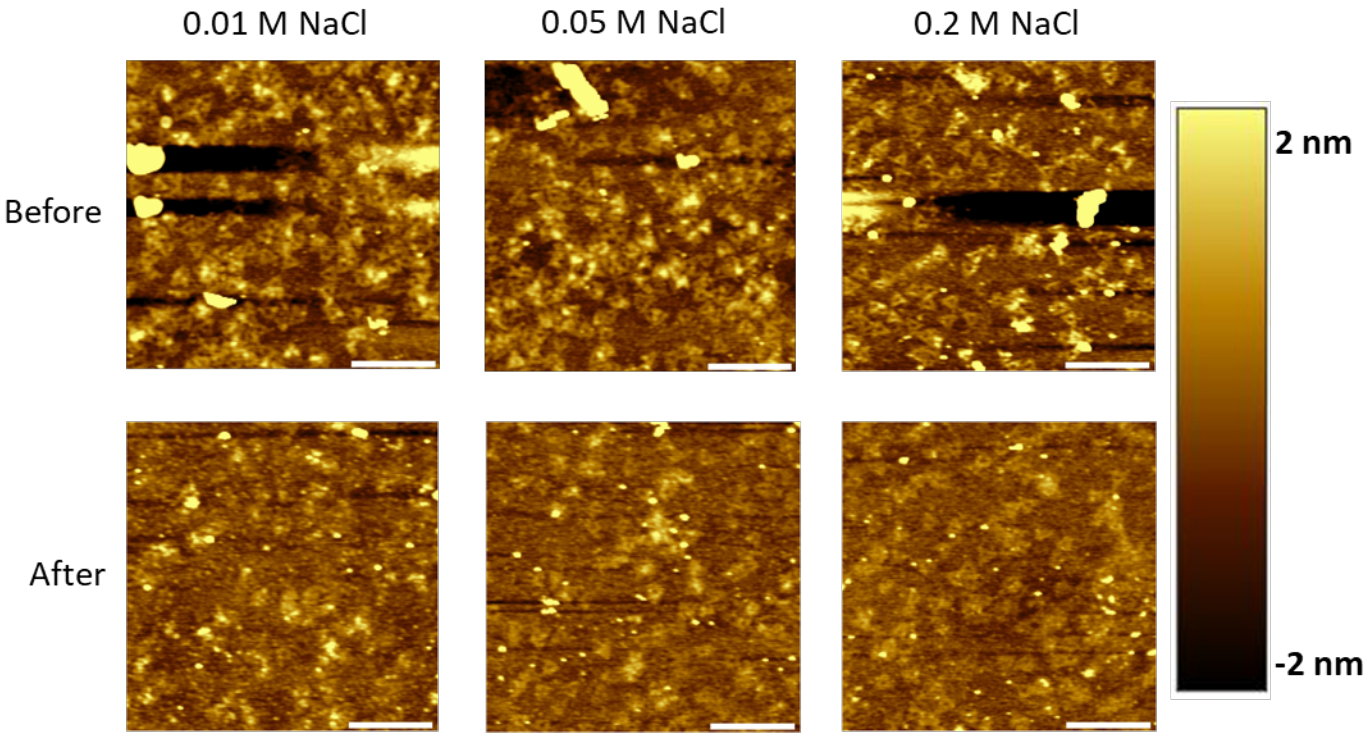

Fig S5. AFM images of DNA origami deposited on APTES before (first row) and after (second row) exposure to 0.01 M NaCl (left column), 0.05 M NaCl (middle column), and 0.2 M NaCl (right column) for 10 sec. All scale bars represent 500 nm.

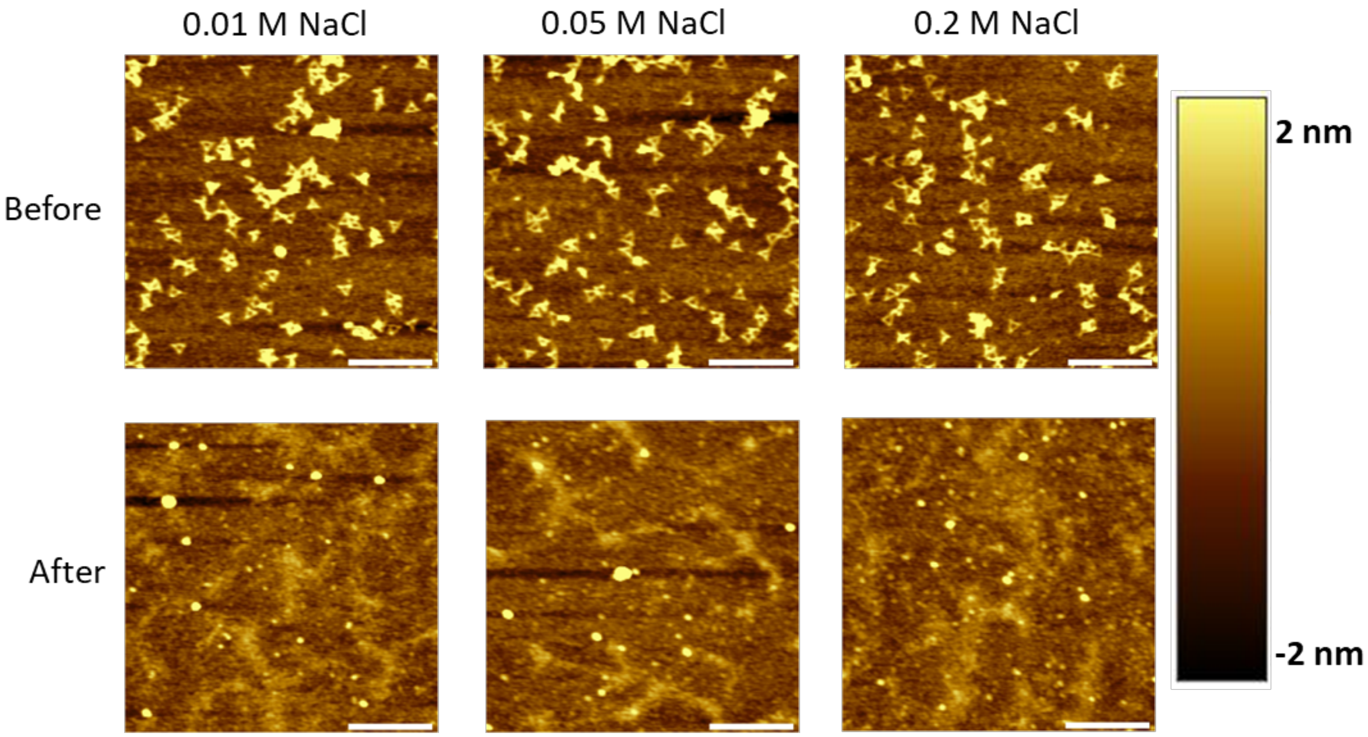

Fig S6. AFM images of DNA origami deposited on PTCS before (first row) and after (second row) exposure to 0.01 M NaCl (left column), 0.05 M NaCl (middle column), and 0.2 M NaCl (right column) for 1 All scale bars represent 500 nm.
